# Pivotal Role of Dogs in Rabies Transmission, China

**DOI:** 10.3201/eid1112.050271

**Published:** 2005-12

**Authors:** Xianchun Tang, Ming Luo, Shuyi Zhang, Anthony R. Fooks, Rongliang Hu, Changchun Tu

**Affiliations:** * Chinese Academy of Science, Beijing, People's Republic of China; † Academy of Military Medical Sciences, Changchun, People's Republic of China; ‡ Veterinary Laboratories Agency (Weybridge), Surrey, United Kingdom

**Keywords:** China, rabies, dog, vaccination, epidemic, zoonosis, dispatch

## Abstract

The number of dog-mediated rabies cases in China has increased exponentially; the number of human deaths has been high, primarily in poor, rural communities. We review the incidence of rabies in China based on data from 1950 and 2004, obtained mainly from epidemiologic bulletins published by the Chinese Ministry of Health.

Rabies is a zoonotic disease that causes severe destruction to the central nervous system and is usually fatal. Asia reports the highest global incidence: human rabies cases there account for >80% of the worldwide total. In Bangladesh, India, and Pakistan, >40,000 persons die of rabies each year; transmission from a dog bite is reported in 94% to 98% of cases (*1*). The numbers of human cases are still considered to be conservative estimates, however, since underreporting of rabies is widespread in developing countries. In recent years, China has reported the second highest rates of illness and death from human rabies worldwide. From 1950 to 2004, ≈103,200 persons died of rabies throughout the country in 4 reported epidemic waves that occurred at 10-year intervals: 1956–1957, 1965–1966, 1974–1975, and 1982–1983. The most severe epidemic occurred from 1980 to 1990 and resulted in 55,367 human deaths (*2*). After 1990, the number of reported human rabies cases declined annually, and the lowest number of cases was reported in 1996 (n = 159), largely as a result of increased awareness of risk. Since 1997, the fatality rate has increased exponentially (*3*); the number of human rabies deaths peaked in 2003–2004. The reported number of human rabies deaths each year from 2001 to 2004 were 854, 1,159, 1,980, and 2,651, respectively, which corresponds to increases of 91%, 36%, 71%, and 34% from the previous year. In addition, the number of affected regions has expanded rapidly, and in 2003, human rabies cases were reported in 190 counties, of which 30 recorded their first epidemic. The current trend shows that the fifth epidemic wave of rabies that began in the 1990s is gaining momentum as a serious human epidemic.

The disease is predominantly distributed in the southern provinces of China, bordered by the Yangtse River. Relatively fewer cases occur in northern China, largely as a result of population demographics. The human-to-dog ratio in southern China is substantially greater than in northern China, and the potential risk for exposure to a rabid dog is therefore enhanced. From 1996 to 2002, rabies predominantly affected 5 southern provinces, Guangxi, Hunan, Jiangxi, Guangdong, and Jiangsu, with human deaths accounting for >70% of the national total (Figure, Table) (*3**–**8*). In 2003, 7 provinces reported twice the number of human rabies cases than reported in 2002. Further analysis showed that the death rate in men was 1–2.5 times higher than in women and that the death rate for adolescents and children was higher than that for adults; 68.6% rabies cases were reported in patients <30 years of age (*5**–**8*). These data reaffirmed our understanding of rabies in other developing regions, especially in parts of Africa and Asia, where rabies is universally recognized as a disease of poor, rural communities, often of the disadvantaged, and principally of young adults and children.

**Figure:**
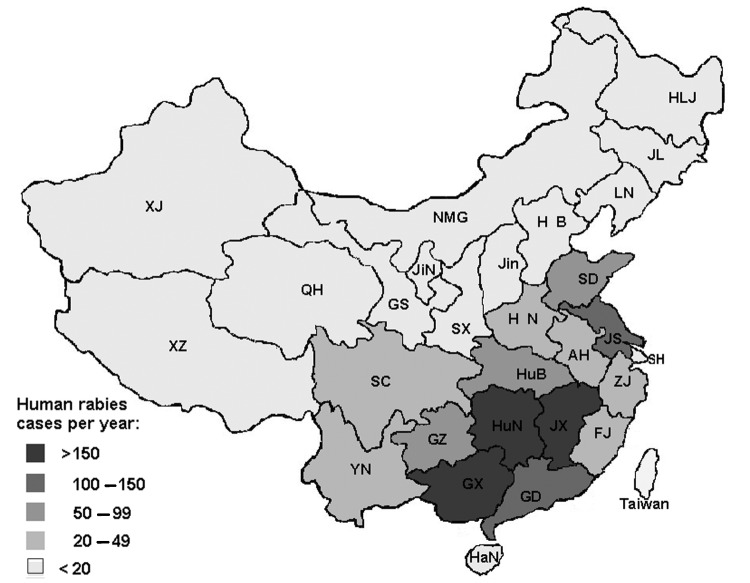
Human rabies epidemic in China by location, 1996–2000.

**Table:** The 5 Chinese provinces with the highest number of rabies cases, 1996–2002

Province	1996	1997	1998	1999	2000	2001	2002
Jiangsu	11	23	15	39	42	48	100
Jiangxi	8	28	60	79	160	192	160
Hunan	17	31	43	78	111	311	300
Guangdong	11	20	29	35	14	76	144
Guangxi	51	49	40	23	79	138	204
Total of 5 provinces	98	151	187	254	406	765	908
National total	163	230	238	341	465	891	1,191
% of national total	60.12	65.65	78.57	74.49	87.31	85.86	76.24

Numerous wildlife species are natural reservoirs of rabies virus and are known, on rare occasions, to act as a source of transmission to humans. In China, however, the domestic dog (*Canis familiaris*) plays a pivotal role in rabies transmission; 85%–95% of human rabies cases are ascribed to dog bites, and 50%–70% of human rabies cases are reported in rural areas. Animal rabies surveillance in 2004 showed that brain tissue specimens collected from 5 (1.76%) of 283 healthy looking dogs from rural areas of 13 cities in Guangxi province tested positive for rabies virus by reverse-transcriptase polymerase chain reaction followed by virus isolation (Q. Liu, pers. comm.). In early 2005, 6 dog rabies cases in rural areas of 5 counties of Chongqing, southwest China, were reported to our laboratory. These rabid dogs were not vacinated and had bitten 15 dogs and 52 people. From their brain tissues 6 rabies virus isolates were obtained.

Owned dogs do not have to be registered in China, and the number of dogs has been estimated at 80–200 million. In rural areas, low vaccination coverage of dogs is widespread, largely because of poor awareness of rabies and the high cost of vaccination. Rural dogs are not leashed and always have free movement in these regions, thereby increasing the risk for human exposure to rabies. More importantly, people injured by dog bites in rural areas do not receive qualified and sufficient postexposure prophylaxis as recommended by the World Health Organization because rabies immunoglobulin is expensive, awareness of prophylaxis is poor, and access to medication is not convenient. One study showed that 244 (88.7%) of 275 human rabies patients reported in Guangxi Province from 1996 to 2000 were not treated with immediate postexposure prophylaxis. Only 31 patients (11.3%) were treated properly; 16 received a completed vaccination regimen (*6*).

In addition to the prevalence of rabies in dogs, the disease was also reported in other domestic animals, livestock, and wildlife, including cattle, pigs, sheep, foxes, sika deer, rats (*9**–**11*), and bats. In 1999, a total of 300 bats not identified to species were captured in Nanning, the capital city of Guangxi province, and their brain tissues were subjected to reverse transcription–polymerase chain reaction and virus isolation to detect a bat lyssavirus. The brain tissues of 3 bats tested positive, resulting in the isolation of live virus by using the mouse inoculation test (T. Luo, pers. comm.). In 2002, the first report of human rabies in China caused from the bite of a bat was reported. A staff member at a television broadcasting station in Tonghua County in northeast Jilin Province was bitten by a bat on the left side of his face when he picked up a telephone on the evening of July 17, 2002. Twelve days later, on July 29, he reported that his face felt numb, and he had a severe headache. On July 31, the patient had a fever with a body temperature of 41°C–42°C. He also reported feeling nauseated and faint and said his upper body was painful and sore. The patient also showed characteristic clinical symptoms of rabies, including a fear of both wind and light. He was hospitalized in Changchun General Hospital, Jilin Province, on August 1, 2002, with a clinical diagnosis of rabies and died the next day, 16 days after the exposure and 4 days after he showed clinical symptoms. This was the first reported case of bat rabies virus in China although the species of the bat was never identified; the tissue samples from the bat and the viral isolates were discarded.

Comprehensive research studies have not been carried in China on the ecology, molecular epidemiology, and genetic diversity of rabies virus strains circulating in different provinces. In 2002, a study demonstrated that the viruses isolated in China from humans and domestic animals were genotype 1 strains of classic rabies virus (*12*).

The prevention and control of rabies would be advanced with the establishment of a veterinary administration that specializes in rabies control. This administration would need financial resources to support diagnostic, surveillance, and vaccination campaigns in animals.

Vaccinating domestic dogs in rural areas would substantially reduce the numbers of human rabies cases. For this goal to be achieved, government-funded registration and licensing for all dogs would have to be compulsory and vaccination and sterilization of owned dogs in rural areas would have to be implemented and regular vaccination of dogs in urban areas continued. If the medical infrastructure in rural areas is strengthened by educating more professional healthcare workers and improving the availability of biological products, especially vaccines and rabies immunoglobulin for human use in postexposure prophylaxis regimens, China would be able to realize the goal of the World Health Organization to reduce by half the number of human rabies cases worldwide by 2015.
